# Effects of pro-inflammatory cytokines on expression of kynurenine pathway enzymes in human dermal fibroblasts

**DOI:** 10.1186/1476-9255-8-25

**Published:** 2011-10-08

**Authors:** Linnéa Asp, Anne-Sofie Johansson, Amandeep Mann, Björn Owe-Larsson, Ewa M Urbanska, Tomasz Kocki, Magdalena Kegel, Göran Engberg, Gabriella BS Lundkvist, Håkan Karlsson

**Affiliations:** 1 Department of Neuroscience, Karolinska Institutet, Retzius väg 8, 171 77 Stockholm, Sweden; 2 Department of Clinical Neuroscience, Karolinska Institutet, Section of Psychiatry at Karolinska University Hospital Huddinge, 141 86 Stockholm, Sweden; 3 Department of Experimental and Clinical Pharmacology, Medical University, Lublin, Jaczewskiego 8, 20-090 Lublin, Poland; 4 Department of Toxicology, Institute of Agricultural Medicine, Lublin, Jaczewskiego 2, 20-950 Lublin, Poland; 5 Department of Physiology and Pharmacology, Karolinska Institutet, Nanna Svartz väg 2, 171 77 Stockholm, Sweden

**Keywords:** human, fibroblast, kynurenine pathway, gene expression, cytokine

## Abstract

**Background:**

The kynurenine pathway (KP) is the main route of tryptophan degradation in the human body and generates several neuroactive and immunomodulatory metabolites. Altered levels of KP-metabolites have been observed in neuropsychiatric and neurodegenerative disorders as well as in patients with affective disorders. The purpose of the present study was to investigate if skin derived human fibroblasts are useful for studies of expression of enzymes in the KP.

**Methods:**

Fibroblast cultures were established from cutaneous biopsies taken from the arm of consenting volunteers. Such cultures were subsequently treated with interferon (IFN)-γ 200 U/ml and/or tumor necrosis factor (TNF)-α, 100 U/ml for 48 hours in serum-free medium. Levels of transcripts encoding different enzymes were determined by real-time PCR and levels of kynurenic acid (KYNA) were determined by HPLC.

**Results:**

At base-line all cultures harbored detectable levels of transcripts encoding KP enzymes, albeit with considerable variation across individuals. Following cytokine treatment, considerable changes in many of the transcripts investigated were observed. For example, increases in the abundance of transcripts encoding indoleamine 2,3-dioxygenase, kynureninase or 3-hydroxyanthranilic acid oxygenase and decreases in the levels of transcripts encoding tryptophan 2,3-dioxygenase, kynurenine aminotransferases or quinolinic acid phosphoribosyltransferase were observed following IFN-γ and TNF-α treatment. Finally, the fibroblast cultures released detectable levels of KYNA in the cell culture medium at base-line conditions, which were increased after IFN-γ, but not TNF-α, treatments.

**Conclusions:**

All of the investigated genes encoding KP enzymes were expressed in human fibroblasts. Expression of many of these appeared to be regulated in response to cytokine treatment as previously reported for other cell types. Fibroblast cultures, thus, appear to be useful for studies of disease-related abnormalities in the kynurenine pathway of tryptophan degradation.

## Introduction

The kynurenine pathway (KP) is the main route of tryptophan degradation in the human body and generates several neuroactive and immunomodulatory metabolites [1,2]. KP activity has the potential to affect a range of neurotransmitter systems in the brain including glutamatergic, cholinergic and serotonergic transmission [2-4]. Indeed, altered levels of KP-metabolites have been observed in neuropsychiatric and neurodegenerative disorders [5-8] as well as in patients with affective disorders [9-13]. While experimental studies support an involvement of kynurenine metabolites in the pathogenesis of both psychiatric and neurodegenerative disorders [14-20], the underlying cause of the dysregulation of kynurenine metabolism in these disorders is not known.

Several studies have shown that infections activate the KP, which thereby appear to serve both as a direct defense mechanism and as a means of modulating the immune response [1,21]. The enzyme indoleamine 2,3-dioxygenase (IDO1) is the first and rate-limiting step of this pathway and is highly induced by the pro-inflammatory cytokine interferon (IFN)-γ [22,23]. However, it is not clear if pro-inflammatory cytokines affect expression of genes encoding other enzymes of the KP. While human fibroblasts have previously been employed for studying the role of IDO1 in controlling experimental infections [24-26], expression or functionality of genes encoding downstream enzymes in the KP have not been investigated in such cells. Since alterations in the KP may potentially reflect the pathophysiology of several neuropsychiatric disorders, it is of major importance to study the KP in primary cells obtained from humans. In the present study, we have established human *ex vivo *skin fibroblast cell cultures as a successful approach to study the KP. We investigated if transcripts encoding enzymes in the kynurenine pathway can be detected in these cells and if their relative abundances are modulated by IFN-γ and/or tumor necrosis factor (TNF)-α.

## Materials and methods

### Tissue isolation and culture

To establish fibroblast cultures, a cutaneous biopsy was taken from the arm of seven consenting volunteers recruited at Karolinska University Hospital Huddinge. Biopsies were minced and placed in 35 mm dishes (Corning Incorporated, Corning NY, USA) under a sterile glass coverslip and cultured in DMEM Glutamax, 10 mM HEPES, 1X MEM amino acids, 1X sodium pyruvate supplemented with 100 U/ml penicillin, 100 μg/ml streptomycin, 15% fetal calf serum (all from Invitrogen, Paisley, UK), in a humidified 37°C, 5% CO_2_ incubator. The regional ethics committee approved the study (04-273/1, supplements 2006/637-32 and 2009-06-12).

### Cytokine treatment

After 2 passages, cells were seeded into 6-well plates (Corning Inc.). At confluence, cytokine treatment was performed during 48 hours using human recombinant TNF-α 100 U/ml or IFN-γ 200 U/ml (PeproTech, London, U.K.) in serum-free media, otherwise as above. Experiments were ended by removal and freezing of the supernatants and addition of lysis buffer to the cell monolayer, see below.

### RNA extraction and reverse transcription

Total RNA was extracted from the cells using the RNeasy Mini kit (Qiagen, GmbH, Hilden, Germany). The amount and purity of the RNA was assessed by spectrophotometry using a Nanodrop ND-1000 (NanoDrop Technologies, Wilmington, DE, USA). Total RNA (250 ng) was subsequently treated with 1 unit of amplification grade DNase I (Invitrogen) for 15 min at room temperature and inactivated by the addition of 2.5 mM EDTA followed by incubation at 65°C for 10 min according to the manufacturer's instructions. The DNase-treated RNA was subsequently reverse transcribed in 20 μl reactions containing the following reagents from Invitrogen; 250 ng of Oligo(dT) primer, 1 × First Strand Buffer, 10 mM DTT and 500 μM of each dNTP and 100 U Superscript II. cDNA synthesis was allowed to proceed for 1 h at 42°C before inactivation at 72°C for 10 min.

### Real-time PCR and data analysis

One μl cDNA templates were added to triplicate 25 μl reaction mixtures using Platinum SYBR Green qPCR Supermix UDG (Invitrogen). An ABI Prism 7500 real-time thermocycler was used (Applied Biosystems, Palo Alto, CA, USA). Primers (Invitrogen) are provided in Table 1. Threshold cycle (Ct) values from the exponential phase of the PCR amplification plot for each target transcript were normalized to that encoding glyceraldehyd-3-phosphate dehydrogenase (GAPDH). From these values, fold-differences in the levels of transcripts between individual untreated and treated cell cultures were calculated according to the formula 2^-ΔΔCt ^[27].

**Table 1:** Transcripts analyzed by real-time PCR, gene symbols and primer sequences

Target transcript	Gene	Polarity	Sequence (5'→3')
IDO1	*INDO*	Sense	GCATTTTTCAGTGTTCTTCGCATA
		Anti-sense	CATACACCAGACCGTCTGATAGCT
TDO	*TDO2*	Sense	GAACATCTTTTTATCATAACTCATCAAGCT
		Anti-sense	ACAACCTTAAGCATGTTCCTTTCAT
KMO	*KMO*	Sense	TGTAATCCTCCAAGCTTCAATCTG
		Anti-sense	CTAGTAGATGCCCACTGAATATTTGTG
HAAO	*HAAO*	Sense	GGACGTTCTGTTTGAGAAGTGGTT
		Anti-sense	AGCTGAAGAACTCCTGGATGATG
KAT1	*CCBL1*	Sense	CCTGCTAAGGCTCAGGTATAACCT
		Anti-sense	GGACTCAAGCCTAAAGGCAACTC
KAT2	*AADAT*	Sense	CACATCTGGCAGCCAACAAG
		Anti-sense	CACTGGCAACATTAATAATGTTGCA
KAT3	*CCBL2*	Sense	ACTATCAGCCATCCCCGTTTC
		Anti-sense	AATGAAGCAAAAACGCACAAACT
KAT4	*GOT2*	Sense	TGTGGTGTGCAGCCTCTCAT
		Anti-sense	AAGCCTGAACCCAGCTAGCA
KYNU	*KYNU*	Sense	ACAGGATCTGCCTCCAGTTGA
		Anti-sense	TGGCCCACTTATCTAGTTCTTCTTC
QPRT	*QPRT*	Sense	ACACCGGCCATGGGTTAAC
		Anti-sense	GCCCCATTGGCCACTGA
GAPDH	*GAPDH*	Sense	CACATGGCCTCCAAGGAGTAA
		Anti-sense	TGAGGGTCTCTCTCTTCCTCTTGT

### Analysis of kynurenic acid levels

Cell culture supernatants (1.0 ml) were collected and kept in -20°C until analysis. In order to precipitate residual protein, samples were centrifuged at 20800 g for 5 minutes and an equal volume of 0.4 M perchloric acid was added to the supernatants. After a second centrifugation 70% perchloric acid (300 μl) was added, and thereafter the supernatants were centrifuged twice at 20800 g for 5 minutes.

Analysis of KYNA was performed using an isocratic reversed-phase high-performance liquid chromatography (HPLC) system, including a dual-piston, high-liquid delivery pump (Bischoff Chromatography, Leonberg, Germany), a ReproSil-Pur C18 column (150 × 4 mm, Dr. Maisch GmbH, Ammerbuch, Germany) and a fluorescence detector (FP 2020, Jasco Ltd., Hachioji City, Japan) with an excitation wavelength of 344 nm and an emission wavelength of 398 nm (18 nm bandwidth). A mobile phase of 50 mM sodium acetate (pH 6.2, adjusted with acetic acid) and 7.0% acetonitrile was pumped through the reversed-phase column at a flow rate of 0.5 mL/min. Samples of 50 μL were manually injected into a Rheodyne injector with a sample loop of 50 μl (Rheodyne, Rhonert Park, CA, USA). Zinc acetate (0.5 M not pH adjusted) was delivered postcolumn by a peristaltic pump (P-500; Pharmacia, Uppsala, Sweden) at a flow rate of 0.10 ml/hr. Signals from the fluorescence detector were transferred to a computer for analysis with Datalys Azur software (Datalys, Grenoble, France). The retention time of KYNA was about 7-8 minutes. Initially, the sensitivity of the system was verified by analysis of a standard mixture of KYNA with concentrations from 1 to 30 nM, which resulted in a linear standard plot.

### Statistics

Comparisons across treatments were done by repeated measures ANOVA with Bonferroni's Multiple Comparison Test using GraphPad (GraphPad Software, Inc., San Diego, CA, USA).

## Results

### Detection of transcripts encoding KP enzymes

**Figure 1:**
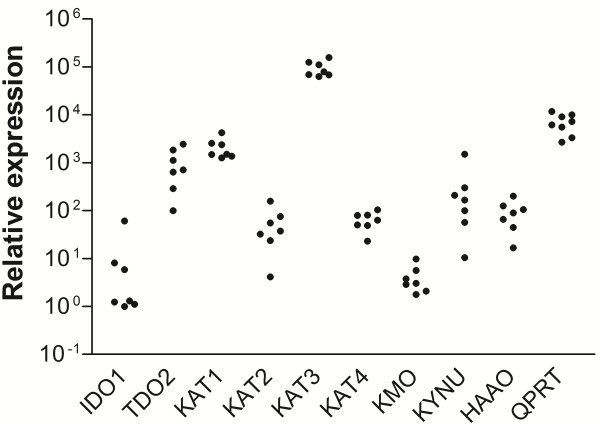
**Relative levels of transcripts encoding enzymes in the kynurenine pathway in human skin-derived fibroblasts from 7 individuals**. Transcripts encoding the following enzymes were investigated; Indoleamine 2,3-dioxygenase 1 (IDO1), Tryptophan 2,3-dioxygenase (TDO), Kynurenine aminotransferases (KAT) 1-4, Kynurenine 3-monooxygenase (KMO), Kynureninase (KYNU), 3-Hydroxyanthranilic acid oxygenase (HAAO) and Quinolinic acid phosphoribosyltransferase (QPRT).

All the investigated kynurenine pathway transcripts (IDO1, TDO, KAT1, KAT2, KAT3, KAT4, KMO, KYNU, HAAO, QPRT) were detected in untreated fibroblast cell cultures, Figure 1. The levels of expression varied considerably across the different genes, with transcripts encoding IDO1 detected at the lowest level and those encoding KAT3 detected at the highest level (difference 8 × 10^3 ^fold). The variation across individual cultures (n = 7), ranged from 2.5 (KAT3) to 145-fold (KYNU).

### Modulation of transcript-levels by IFN-γ and/or TNF-α

**Figure 2:**
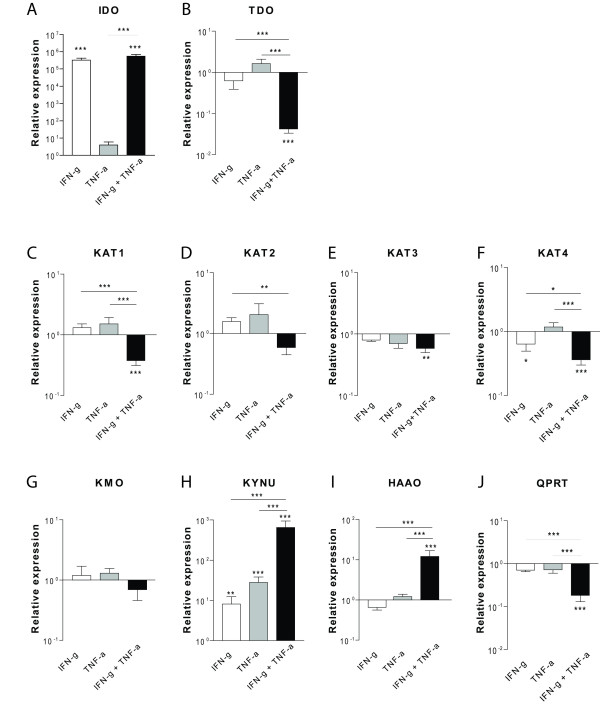
**Relative levels of transcripts encoding enzymes in the kynurenine pathway (A-J) following treatment with IFN-γ (200 U/ml), TNF-α (100 U/ml) or the combination of these two cytokines (IFN-γ+TNF-α) during 48 hrs in serum-free cell culture medium (n = 7)**. Levels of all transcripts are normalized to levels observed in untreated control cells (base-line). *p < 0.05, **p < 0.01, ***p < 0.001.

The potential effects of IFN-γ, TNF-α, or a combination of IFN-γ and TNF-α on kynurenine pathway transcripts were investigated in the fibroblast cell cultures, see Figure 2. The levels of transcripts encoding IDO1 were significantly increased (> 10^5^-fold) in cultures treated with IFN-γ (p < 0.001) as well as IFN-γ together with TNF-α (p < 0.001) compared to untreated cultures although no effect of TNF-α alone was observed (Figure 2A). Transcripts encoding tryptophan 2,3-dioxygenase (TDO), on the other hand, were significantly down-regulated in cultures treated with a combination of IFN-γ and TNF-α (20-fold; p < 0.001) as compared to untreated cells or cells treated with the individuals cytokines (Figure 2B). Moreover, levels of transcripts encoding the kynurenine aminotransferases (KATs) were either unaffected or down-regulated by the cytokine treatments. Whereas KAT2 was unaffected by cytokine treatment, KAT1 and KAT3 transcript levels were reduced following treatment with the combination of IFN-γ and TNF-α (2.6-fold, p < 0.001 and 1.7-fold, p < 0.01 respectively, Figure 2C, D and 2E). Levels of transcripts encoding mitochondrial aspartate aminotransferase (mitAAT, i.e KAT4) were significantly down regulated (1.5-fold) in cultures treated with IFN-γ (p < 0.05) and further decreased with the combination of IFN-γ and TNF-α (2.7-fold; p < 0.001, Figure 2F). Levels of transcripts encoding kynurenine 3-monooxygenase (KMO) observed in the fibroblast cultures were not significantly affected by the cytokine treatment (Figure 2G). Levels of transcripts encoding kynureninase (KYNU) were up-regulated following IFN-γ treatment (8-fold; p < 0.01) or with TNF-α treatment (28-fold; p < 0.001). A further increase in the levels of KYNU transcripts was observed with the combination of IFN-γ and TNF-α (650-fold; p < 0.001, Figure 2H). Levels of transcripts encoding 3-hydroxyanthranilate 3,4-dioxygenase (HAAO) were up regulated only in cultures treated with the combination of IFN-γ and TNF-α (12-fold, p < 0.001, Figure 2I). Levels of transcripts encoding quinolinate phosphoribosyltransferase (QPRT) were down-regulated by the combination of IFN-γ and TNF-α (5-fold, p < 0.001), but unaffected by the individual cytokines (Figure 2J).

### Effects on KYNA levels

To address potential functionality of the KP in these human fibroblast cultures, we measured the accumulation of KYNA, one of the end metabolites in the KP in the supernatants. Levels of KYNA were detectable in supernatants from untreated *ex vivo *fibroblast cultures (3.4 ± 0.6 nmol/l). Significantly (p < 0.0001) higher levels were detected in supernatants of cells treated with IFN-γ (27.2 ± 18 nmol/l) or with IFN-γ and TNF-α (39.8 ± 20.1 nmol/l) as compared to supernatants from untreated cells. TNF-α alone did not cause a significant increase in the accumulation of KYNA.

## Discussion

We here report, for the first time, that human skin fibroblast cultures express detectable levels of transcripts encoding the different enzymes of the KP. Substantial differences in the basal levels of expression across genes and individuals were observed which are likely to be explained by genetic and epigenetic variation between individual cultures. Following treatment with IFN-γ, these cultures exhibited relative increases of > 10^5^-fold for transcripts encoding IDO1. We also found that human skin fibroblast cultures can release KYNA, and that this release was significantly increased following IFN-γ, but not TNF-α, treatment, indicating that at least some of the transcriptional changes observed in response to IFN-γ are functional in these cells.

Thus, in agreement with previous reports [28,29], human fibroblast cultures appear to be able to increase the rate of tryptophan degradation along the kynurenine pathway in response to IFN-γ treatment. Our present findings support the notion that IDO1 is the major determinant of this response in human fibroblasts, as is also the case in many other cell types, derived both from the brain and from peripheral tissues [30]. For example, Guillemin and co-workers reported increased levels of KYNA and increased levels of transcripts encoding IDO1 following IFN-γ, but not following TNF-α treatment of human fetal astrocytes [23]. More recently, increased levels of KYNA and transcripts encoding IDO1 were also observed in primary neurons and neuroblastoma cells following IFN-γ treatment [22].

While Heyes and colleagues [31] reported a small increase in KMO activity in monocytes following IFN-γ treatment, we did not observe any significant effect on transcripts encoding KMO following cytokine treatment. Our observations are thus in agreement with the effects of IFN-γ observed in neuronal cells [22]. Whereas IFN-γ or TNF-α, alone or in combination, markedly increased transcripts of KYNU and HAAO, we observed no effect or even decreased levels of transcripts encoding KAT enzymes by these cytokines. Indeed there is no consensus in earlier studies regarding the response of the KAT enzymes to IFN-γ treatment. Whereas increases in the levels of KAT 1 and KAT 2 were observed in fetal astrocytes following IFN-γ treatment [23], no effect on the levels of transcripts encoding these enzymes was observed in neuronal cells [22]. In neuroblastoma cells, levels of transcripts encoding TDO were reduced by the IFN-γ treatment whereas no effects on the levels of transcripts encoding KAT1, KAT2, KYNU, KMO, HAAO or QPRT were observed [22]. Differences in transcription of genes encoding enzymes involved in the KP in response to IFN-γ therefore most likely exist across cell types. These differences probably also explain some of the differences observed across cell types in their enzyme activities and in their abilities to form kynurenine and quinolinic acid [31]. The physiological role of the kynurenine pathway in skin-derived fibroblasts is not known but may involve effects not primarily related to acetylcholine or glutamate receptors such as effects on cell proliferation [1], cytokine release [32] or microbial growth [21,24-26,33] as described in other peripheral cell types.

**Figure 3:**
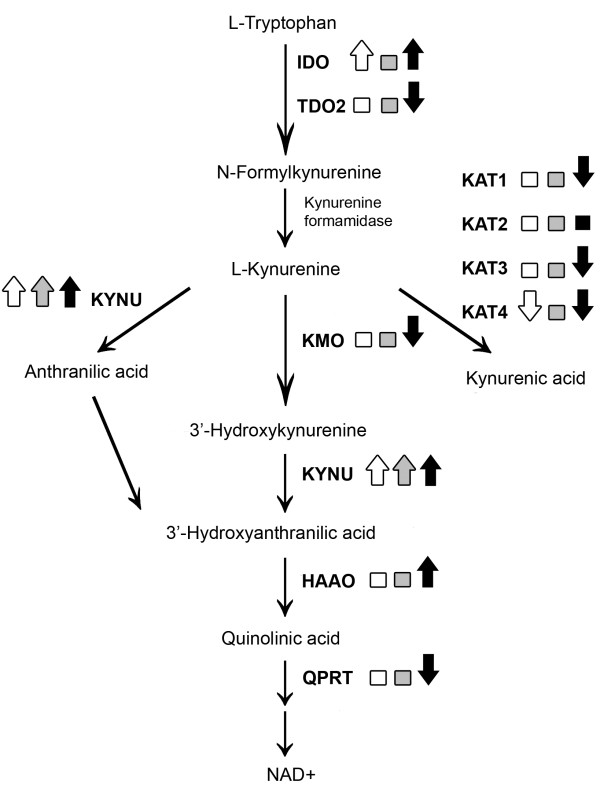
**Overview of the changes in levels of transcripts encoding enzymes in the kynurenine pathway in human skin-derived fibroblasts following treatment with IFN-γ (white), TNF-α (gray) or the combination of these two cytokines (black) during 48 hrs**. Squares indicate no significant change whereas arrows indicate significant up- or down-regulation.

The increases in KYNU and HAAO, and decrease in levels of transcripts encoding QPRT, following IFN-γ and TNF-α treatment suggest that such treatment can potentially alter the accumulation of other metabolites generated by the KP, such as quinolinic acid. It should also be noted that TNF-α treatment alone caused a pronounced and selective increase (almost 30-fold) in levels of transcripts encoding KYNU, suggesting a direct influence of TNF-α on expression of this gene. Thus, it appears as if certain cytokines can differentially affect expression of genes in the KP, at least in fibroblasts (for overview see Figure 3), and thereby potentially modulate levels of individual metabolites.

Fibroblast cultures derived from patients and healthy controls have previously been used to study a range of CNS-diseases. For example, in fibroblasts from patients with schizophrenia, alterations in pathways involved in cell cycle regulation and RNA processing have been identified [34]. Moreover, alterations in growth, morphology, cell adhesion, apoptotic pathways, composition of phospholipid fatty acids in the plasma membrane and glutathione synthesis are reported [35-39]. Aberrant amino acid transport has been identified in fibroblast from patients with schizophrenia, bipolar disorder as well as autism [40-42]. These reports suggest that peripheral tissues can be used to identify alterations at the molecular level in patients with psychiatric disorders and thus provide a useful method to investigate mechanisms underlying such disorders. The advantage of studying *ex vivo *cultures compared to *postmortem *tissue or blood samples is that in such cultures confounding factors like medical treatments are minimized. Furthermore, in contrast to using clinical samples, *ex vivo *cell cultures can also be used to conduct well-controlled studies of potential gene-environment interactions. The present findings suggest that fibroblast cultures can be used to study disease-related abnormalities in the kynurenine pathway of tryptophan degradation.

## Competing interests

The authors declare that they have no competing interests.

## Authors' contributions

BOL performed biopsies. ASJ performed cell cultures. LA and AM carried out the RNA analyses. MK carried out the KYNA analyses. LA performed all statistical analyses. EMU, TK participated in the design of the study. HK, GE, GSL and EMU conceived of the study, and participated in its design and coordination. HK drafted the manuscript. All authors helped to revise the first draft of the manuscript and all authors approved the final manuscript.
